# Coxsackievirus Group B Infections during Pregnancy: An Updated Literature Review

**DOI:** 10.3390/jcm13164922

**Published:** 2024-08-21

**Authors:** Carolina Longo, Mauricio Saito, Pedro Teixeira Castro, Evelyn Traina, Heron Werner, Julio Elito Júnior, Edward Araujo Júnior

**Affiliations:** 1 Department of Obstetrics, Paulista School of Medicine, Federal University of São Paulo (EPM-UNIFESP), São Paulo 04023-062, SP, Brazil; carolinalongo1989@gmail.com (C.L.); evelyntraina@gmail.com (E.T.); elitojjr@hotmail.com (J.E.J.); 2 CONCEPTUS—Fetal Medicine Center, São Paulo 04001-084, SP, Brazil; mauriciosaito@uol.com.br; 3 Department of Fetal Medicine, Biodesign Laboratory DASA/PUC, Rio de Janeiro 22451-900, RJ, Brazil; pedrotcastro@gmail.com (P.T.C.); heron.werner@gmail.com (H.W.); 4 Discipline of Woman Health, Municipal University of São Caetano do Sul (USCS), São Caetano do Sul 09521-160, SP, Brazil

**Keywords:** coxsackievirus group B, maternal infection, vertical transmission, fetal malformation, perinatal outcomes

## Abstract

Coxsackievirus group B (CVB), a member of the *Picornaviridae* family and enterovirus genus, poses risks during pregnancy due to its potential to cause severe fetal and neonatal infections. Transmission primarily occurs through fecal–oral routes, with infections peaking mostly in warmer months. Vertical transmission to the fetus can lead to conditions such as myocarditis, encephalitis, and systemic neonatal disease, presenting clinically as severe myocardial syndromes and neurological deficits. Diagnostic challenges include detecting asymptomatic maternal infections and conducting in utero assessments using advanced techniques like RT-PCR from amniotic fluid samples. Morbidity and mortality associated with congenital CVB infections are notable, linked to preterm delivery, fetal growth restriction, and potential long-term health impacts such as type 1 diabetes mellitus and structural cardiac anomalies. Current treatments are limited to supportive care, with emerging therapies showing promise but requiring further study for efficacy in utero. Preventive measures focus on infection control and hygiene to mitigate transmission risks, which are crucial especially during pregnancy. Future research should aim to fill knowledge gaps in epidemiology, improve diagnostic capabilities, and develop targeted interventions to enhance maternal and fetal outcomes.

## 1. Introduction

Coxsackievirus group B belongs to the family *Picornaviridae* and the genus enterovirus. Enteroviruses (EVs) are positive-sense, single-stranded RNA viruses, named for their gastrointestinal route of transmission [1]. These viruses are categorized based on their pathogenesis in humans and laboratory animals into four groups: polioviruses, coxsackie A viruses (CA), coxsackie B viruses (CB), and echoviruses [2].

There are at least twenty-three recognized serotypes of group A and six serotypes of group B: CV-B1, CV-B2, CV-B3, CV-B4, CV-B5, and CV-B6 [3]. Humans are the only known reservoir for coxsackieviruses A and B, which spread through fecal–oral and possibly respiratory routes [4].

Group A coxsackieviruses typically cause flaccid paralysis due to generalized myositis. In contrast, group B coxsackieviruses are associated with spastic paralysis, resulting from neuronal tissue degeneration and focal muscle injury. Additionally, group B coxsackieviruses can infect the heart, pleura, pancreas, and liver, leading to conditions such as pleurodynia, myocarditis, pericarditis, and hepatitis [5]. They are also known to cause systemic neonatal infection, characterized by multiorgan involvement, one of the most potentially fatal conditions associated with enterovirus [6,7]. This review will focus on the coxsackievirus subgroup B.

In this literature review, we will explore key aspects of coxsackievirus group B (CVB) infections during pregnancy. We will discuss the epidemiology and transmission routes of CVB, including vertical transmission risks. The pathophysiology of CVB will be examined, focusing on interactions with cellular receptors and effects on the heart and central nervous system. Diagnostic challenges and methods for detecting maternal and fetal infections will be evaluated. We will also consider the morbidity and mortality associated with congenital infections, including long-term health impacts. Finally, treatment options and preventive measures will be discussed, highlighting the need for further research and improved infection control strategies.

## 2. Materials and Methods

A comprehensive literature search was conducted to gather relevant studies on CVB and its impact on pregnancy. The following databases were systematically searched: PubMed/MEDLINE, Scopus, Web of Science, and Google Scholar. Given the limited literature on this topic, the search included articles published from January 1985 to June 2023. The search terms used included combinations of the following keywords: “Coxsackie B virus”, “CVB”, “pregnancy”, “maternal infection”, “fetal outcomes”, “neonatal infection”, “vertical transmission”, and “congenital infection”.

### 2.1. Epidemiology

Coxsackievirus group B affects both males and females equally and can occur worldwide, with infections typically peaking during the warm summer months. Frequently affecting children under one year of age [8], enteroviral infections are commonly found in late pregnancy, particularly during times when these infections are widespread in the community [9].

Often, enteroviral infections go unnoticed, even during pregnancy. However, some case histories and mouse experiments suggest that these viruses can be transmitted vertically. More commonly, transmission occurs through fecal contamination during and shortly after birth [10].

Emerging clinical evidence indicates that intrauterine coxsackievirus B (CVB) infection during late gestation or the perinatal period can lead to severe, life-threatening diseases such as neonatal myocarditis, meningitis, hepatitis, encephalitis, long-term neurological deficits, or sudden death [11]. Additionally, infection during pregnancy may cause preterm delivery, fetal growth retardation, or embryopathy, as suggested by a few case reports [12,13].

### 2.2. Pathophysiology

The pathogenesis of coxsackieviruses (CVs) is predicated on specific interactions between the virus and cellular receptors. These interactions dictate the primary site of infection and subsequent dissemination to other organs during the post-viremic phase [14]. The initiation of CVB infection entails three primary stages: the attachment of infectious virions to specific receptor proteins, internalization and uncoating of virions, and interaction of viral RNA with intracellular factors essential for virus replication and protein synthesis [15].

**Figure 1:**
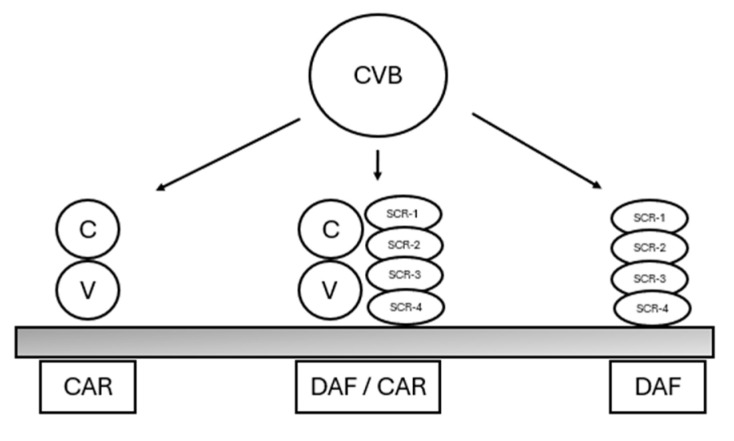
A schematic representation of CVB–receptor interactions. Coxsackie B virus (CVB) primarily binds to two cell surface proteins: the coxsackievirus–adenovirus receptor (CAR) and the decay-accelerating factor (DAF/CD55). These CAR and DAF molecules may collaborate to form a functional DAF/CAR receptor complex. Adapted from Selinka et al. [15].

An analysis of capsid mutations among different CVB strains indicates that virus–receptor interactions play a crucial role in CVB infection pathogenesis [16]. The six serotypes of CVB are known to interact with at least two specific receptor proteins (Figure 1). The primary receptor on cells for CVB is the coxsackievirus–adenovirus receptor (CAR), a 46 kDa protein from the immunoglobulin superfamily, which is also utilized by adenoviruses [17,18]. Specific CVB strains engage with another 70 kDa molecule known as decay-accelerating factor (DAF/CD55), which is a cell surface-expressed protein involved in regulating complement activity [19]. Given that specific receptor proteins determine the cellular and tissue tropism of nonenveloped viruses, the diverse diseases caused by CVB, encompassing acute and chronic myocarditis, aseptic meningitis, and pancreatitis, may reflect variations in interactions with these cellular receptors [15].

In 2014, Young et al. [11] demonstrated the robust expression of the coxsackievirus and adenovirus receptor (CAR) not only in embryos but also in the endometrial epithelia and uterine glands of ICR mice, implying that the uterus is a potential target for CVB. Their findings suggest that the uterus and embryo, both rich in CAR expression, are pivotal targets of CVB3, with vertical transmission during early gestation resulting in pregnancy loss.

CAR has been identified as a crucial molecule for embryo development [20,21,22]. Koi et al. [23] proposed that there is heightened susceptibility to adenovirus infection during the first trimester due to CAR expression in both the villous trophoblast cells and extravillous trophoblast cells of the human placenta at 11 weeks. They further demonstrated CAR expression throughout gestation in extravillous trophoblast cells, susceptible to fiber-mediated adenovirus attachment and subsequent infection-induced apoptosis. The CAR protein and RNA are absent in villous trophoblast cells isolated from third-trimester placentae, despite the efficient transduction of primary villous cytotrophoblast cells by adenovirus vectors [24]. Maternal adenovirus infection early in gestation may lead to trophoblast infection, the transplacental passage of adenovirus, and latent fetal infection [23].

Elevated apoptotic trophoblast cells are linked to placental aging and fetal growth restriction [25,26]. Koi et al. [23] demonstrated that both adenovirus infection and the maternal immune response to infection (decidual lymphocytes) are necessary to induce apoptotic changes in extravillous trophoblast cells. Adenovirus-induced apoptosis may accelerate placental aging and impair extravillous trophoblast function, Adenovirus-induced apoptosis may accelerate placental aging and impair extravillous trophoblast function, which can increase the risk of obstetric complications in pregnant women, such as pre-eclampsia, restricted fetal growth, and fetal loss.

#### 2.2.1. Coxsackievirus Group B and Heart Tissue

DAF is expressed in epithelial and endothelial cells, while CAR is present in intercalated discs linking myocardial cells. Interaction with these receptors facilitates coxsackie B virus entry into myocardial cells, ultimately leading to myocarditis [14,15].

#### 2.2.2. Coxsackievirus Group B and Central Nervous System

Central nervous system (CNS) infections may occur via hematogenous spread or axonal transport, particularly when nerve terminals or muscles are damaged [27,28]. The viral replication or activation of the immune system can lead to CNS tissue injury [29]. Although the precise mechanisms underlying cell injury and death remain elusive, the viral inhibition of cellular macromolecular production, the toxicity of viral proteins, and virus-induced apoptosis are believed to contribute [29,30].

### 2.3. Transmission

Coxsackie viruses and echoviruses are common causes of infection in neonates and infants less than one year old [31,32,33]. The transmission of enterovirus from the mother to the neonate typically occurs through contact with infected maternal secretions during vaginal delivery [34,35].

The transplacental transmission of enteroviruses has also been documented and is generally associated with more severe and often fatal outcomes [36,37]. In such cases, the illness usually manifests within the first few days of life [38]. Pre- or perinatal transmission is known to cause more severe disease compared to postnatal transmission from the mother, family, or hospital staff [9].

Determining the exact route of enterovirus acquisition can be challenging, but the presence of maternal illness and the timing of disease onset can provide clues. If the mother experiences illness within two weeks prior to delivery, she is likely the source of infection. Given that the incubation period of enteroviral disease is typically 3 to 5 days (ranging from 2 to 12 days), the onset of disease within the first two days of life suggests transplacental infection [34].

## 3. Diagnosis

### 3.1. Maternal Diagnosis

Enterovirus infections in adults typically manifest as mild, often unnoticed symptoms. The primary clinical manifestation is an acute, self-limiting febrile illness. Diagnosis in adults poses challenges due to the predominantly asymptomatic nature of the infection and the nonspecific symptoms. Additionally, enterovirus infections are underdiagnosed due to limited awareness among medical professionals. Symptoms in pregnant women closely resemble those in the general adult population and are primarily characterized by fever. However, flu-like symptoms, diarrhea, conjunctivitis, or rash (exanthema, foot-and-mouth syndrome) may also occur [39]. Symptoms can range from myocarditis, pericarditis, and pleurodynia (also known as Bornholm disease) to skin rashes, mouth and throat ulcers, hand-foot-and-mouth disease, herpangina, acute hemorrhagic conjunctivitis, and, in some cases, aseptic meningitis or encephalitis [40].

The biological diagnosis of enterovirus infection relies on specific tests. Indirect enzyme-linked immunosorbent assays (ELISAs) can detect IgG and IgM antibodies against a panel of coxsackieviruses (CVs) in blood samples [41]. However, the most sensitive and specific method is a reverse transcription/polymerase chain reaction (RT-PCR) analysis of blood, throat, cerebrospinal fluid (CSF), or amniotic fluid to detect viral RNA. The interpretation of a positive RT-PCR result depends on the sampling site’s specificity. Positive results in CSF or amniotic fluid are highly significant, whereas stool samples lack specificity for acute infection [42].

Enterovirus presence in the throat is common, even in asymptomatic individuals, making blood samples more specific. RT-PCR for enterovirus is typically positive in maternal blood during the acute phase of infection, often while the patient remains febrile. Although the positivity in blood decreases soon after, it can persist for several days (up to 3 weeks) in stool samples [39].

### 3.2. Fetal Diagnosis

Diagnosing a coxsackie B viral infection in utero is challenging due to the inability to access traditional sample sources such as respiratory secretions, blood, cerebrospinal fluid (CSF), stool, and urine. However, transplacental infection can be inferred when the virus is isolated from the amniotic fluid [37,43,44].

**Figure 2:**
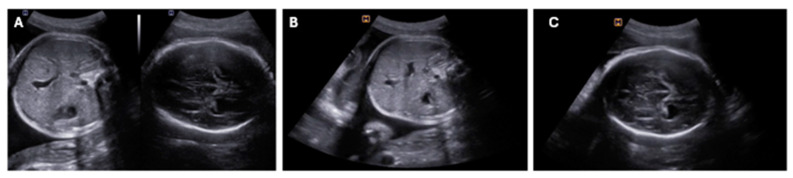
Two-dimensional ultrasound findings in axial views of intrauterine coxsackievirus group B infection. (**A**) Relationship between abdomen and head showing mild fetal growth restriction; (**B**) discrete enlargement of liver and spleen and presence of microcalcifications; (**C**) increased cerebral echogenicity in periventricular and deep white matter (from our own database).

Fetal enterovirus infection can lead to various sonographic findings, including cerebral ventriculomegaly, cardiomyopathy with ventricular dysfunction [45] (Figure 2), polyhydramnios associated with ascites, and pericardial and pleural effusions, which can result in death due to multi-visceral failure [46]. Bonnin et al. [47] reported a case of congenital infection with coxsackie virus B5, manifesting at 34 weeks with symptoms such as decreased fetal movements, polyhydramnios, and myocarditis (Table 1).

Placental tissue examination can reveal signs of intrauterine infection, particularly through the inflammation of the placental villi, which indicates that the infection has spread to the placenta via the maternal bloodstream. A hypothesis for this is that a significant number of macrophages, granulocytes, and lymphocytes typically present in virus-induced villitides cause stem vessel vasculitis and chronic villitis. This inflammatory response results in an acute type of hematogenous placentitis.

This phenomenon has been observed in fatal cases of fetal coxsackie B3 and A9 infections [48]. Viral studies for enterovirus can be conducted, and if positive, subsequent polymerase chain reaction (PCR) testing can specifically identify the presence of coxsackie B4 virus in the amniotic fluid sample [37] (Table 2).

## 4. Morbidity/Mortality

The onset of illness in most fatal coxsackie B and echovirus infections occurs at birth or within the first few days, indicating that transplacental infection carries a worse prognosis. Symptoms appearing after 14 days of age are usually due to horizontal transmission and are associated with less severe disease. The most common neonatal manifestations of these infections include myocarditis [49], meningoencephalitis [50], hepatitis, disseminated intravascular coagulopathy, and sepsis-like syndromes [31,51]. Less common presentations include pneumonia, gastrointestinal symptoms, pancreatitis, cutaneous exanthems, and seizures [33,36]. The combination of cardiomyopathy and coagulopathy is strongly associated with mortality [34].

Congenital infection could be linked to childhood type I diabetes [52,53]. A prospective study conducted in Finland revealed that children who later developed insulin-dependent diabetes mellitus (IDDM) had nearly twice the frequency of intrauterine exposure to coxsackie B virus (CBV) and other enteroviruses, as confirmed by serological tests, compared to their siblings who did not develop diabetes [54]. A comparable study in Sweden retrospectively tested serum samples from 55 women whose infants later developed diabetes for IgM antibodies against coxsackie B2, B3, and B4 viruses. The study found that positive serology for these viruses was three times more frequent compared to a matched control group. However, no significant differences were observed in serology for herpes, mumps, and toxoplasmosis in the same serum samples [52,55].

In contrast, a more recent prospective study by Füchtenbusch et al. [56] in Germany followed 28 infants born to parents with diabetes from birth until the development of islet autoantibodies. They compared these infants with 51 offspring of diabetic parents who did not develop autoantibodies and found no significant difference in the incidence of coxsackie B3, B4, and B5 infections between the two groups. Additionally, there were no discernible variations in maternal serum antibody levels during pregnancy and at birth. These conflicting findings complicate the establishment of a direct connection between maternal coxsackie B infection during pregnancy and fetal or neonatal pancreatic damage leading to type 1 diabetes mellitus. If such a relationship does indeed exist, it appears to be uncommon [40,56].

Congenital infection has also been linked to neurodevelopmental delays [57], structural cardiac anomalies [58], and severe anatomic defects of the central nervous system (CNS) such as hydranencephaly and hydrocephalus [59]. In 2004, Bryant et al. [34] reported on two of ten cases of hepatitis, noting that both patients exhibited severe disease. These cases were characterized by significantly elevated liver enzymes and coagulopathy. In 2001, Abzug [60] identified a correlation between these clinical features and increased mortality in echovirus infections. The case-fatality rate of 31% underscores the potentially devastating impact of enterovirus hepatitis and coagulopathy (EHC), particularly in neonates, highlighting its severe and life-threatening nature.

A 2020 study by Vipul Sharma et al. [61] showed a significant association between in utero coxsackievirus B (CVB) infection/exposure and congenital heart defects, specifically Pulmonary Atresia (PA) and Hypoplastic Left Heart Syndrome with Aortic Atresia/Mitral Stenosis variant (HLHS AA/MS). An interesting observation from the study was that one subject with high CVB IgG levels had a fetus affected by an isolated right ventricular (RV) associated coronary fistula. These findings suggest that fetal exposure to CVB could potentially lead to the formation of abnormal myocardial architecture and associated sinusoids. The authors recommend a large prospective cohort study to further assess these findings.

Research conducted by Satosar et al. [62] revealed that among 60 placentae collected from cases involving fetal or neonatal death or severe neonatal morbidity, coxsackievirus was identified in 22 cases. In contrast, none of the 17 control placentae showed any detectable infectious agent. These findings underscore the potential pathogenicity of coxsackievirus in late-term pregnancies, potentially contributing to perinatal mortality or morbidity.

Axelsson et al. [63] conducted a study in Sweden comparing CVB infection rates during pregnancy between 97 women who experienced miscarriage (80 within the first 12 weeks) and 113 control women who underwent voluntary pregnancy termination at similar gestational weeks. They assessed maternal IgM antibody levels for CVB as an indicator of recent infection during pregnancy. The study revealed a significantly higher prevalence of positive CVB-IgM antibodies among women who miscarried before the 13th week compared to control women. However, there was no difference in CVB infection rates between the groups if miscarriage occurred between 13 and 27 weeks of gestation.

## 5. Treatment

The prenatal diagnosis of intrauterine infection provides an increased opportunity for fetal therapy. Empirical treatments proposed for neonates infected with enteroviruses have included the combined therapy of intravenous gamma or hyperimmune globulin and leukocyte interferon [64]. However, such treatments have not been reported for in utero therapy, and there is no evidence that they can prevent the fatal outcome of the disease [43]. Specific antiviral therapy, such as Pleconaril, shows promise in treating meningitis and other life-threatening infections caused by enteroviruses.

Pleconaril, a specific antipicornavirus agent, has become available for compassionate use in at-risk patients. The preliminary results presented in this report suggest that Pleconaril has beneficial effects on clinical, virological, laboratory, and radiological parameters. Rotbart et al. [65] demonstrated that among the six patients with overwhelming neonatal enterovirus infection in the Pleconaril trial, five survived, including all four who were treated within the first week of illness. The sequelae among the surviving babies were mostly mild. However, the number of treated patients in this compassionate-use study is too small to determine whether the outcome was significantly improved compared to neonates not treated with Pleconaril.

Currently, no treatments or vaccines are available to protect against CVB infection. Therefore, healthcare professionals should emphasize infection control strategies, such as maintaining effective hand and environmental hygiene [66]. Pregnant women should avoid contact with individuals suspected of having an enterovirus infection to prevent severe neonatal disease and perinatal complications [31].

## 6. Prognosis

In adults, enterovirus infections are typically self-limiting, with most cases resulting in full recovery. However, there are a few notable exceptions. While aseptic meningitis caused by enteroviruses generally has an excellent prognosis, some patients may experience prolonged malaise and fatigue for several weeks. In infants and children, aseptic meningitis can lead to mild intellectual complications. Myocardial syndromes, although rare, can sometimes result in poor outcomes [67].

During pregnancy, the outcomes of coxsackievirus B (CVB) infection are influenced by several factors, including the susceptibility of the pregnant individual, the amount of virus reaching the uterus or embryos, and the developmental stage of the embryos. Maternal CVB infection in early pregnancy is particularly associated with a high rate of pregnancy loss [11]. Therefore, further research should prioritize investigating the prevalence of CVB infections in cases of miscarriage. Establishing CVB as a significant causative agent could potentially lead to the development of vaccines aimed at preventing pregnancy loss.

In general, infection with coxsackievirus B (CVB) during pregnancy poses risks to the embryo and fetus, potentially resulting in fetal and neonatal mortality and potentially causing cardiac abnormalities. Specifically, maternal infection late in pregnancy often leads to severe clinical symptoms in newborns. However, the existing literature lacks adequate data to precisely determine the frequency of these fetal complications [40].

## 7. Conclusions

CVB infections during pregnancy present significant risks due to complex pathogenesis involving specific cellular receptors like CAR and DAF, facilitating viral entry and tissue damage. CVB exhibits a widespread distribution, with rare but severe outcomes in vertical transmission leading to conditions such as myocarditis and encephalitis. Diagnostic challenges persist, especially with asymptomatic maternal infections, relying on advanced techniques like RT-PCR. Morbidity includes adverse fetal outcomes like preterm delivery and neurological deficits, with potential links to type 1 diabetes and cardiac anomalies requiring further research. Treatment options are limited to supportive care and unproven antiviral therapies like Pleconaril, emphasizing the importance of preventive measures for transmission control during pregnancy. CVB infections during pregnancy present a serious threat to fetal and neonatal health. While significant strides have been made in understanding the virus’s pathophysiology and transmission, considerable gaps remain in our knowledge, particularly regarding the incidence and long-term impact of congenital infections. Ongoing research and improved diagnostic, therapeutic, and preventive strategies are essential to address this public health concern effectively.

## Figures and Tables

**Table 1:** Clinical maternal and fetal presentation of intrauterine coxsackie B virus infection.

Binomial	Clinical Presentation
Maternal	Asymptomatic Mild nonspecific symptoms Acute, self-limiting febrile illness (flu-like symptoms) Diarrhea Conjunctivitis Rash (exanthema, enanthemas, foot-and-mouth syndrome) Herpangina Myocarditis Pericarditis Pleurodynia (Bornholm disease) Acute hemorrhagic conjunctivitis Aseptic meningitis or encephalitis (occasionally)
Fetal	Cerebral ventriculomegaly Cardiomyopathy with ventricular dysfunction Polyhydramnios Ascites Pericardial and pleural effusions Decreased fetal movements Multi-visceral failure Intrauterine fetal death

**Table 2:** Classification of maternal intrauterine coxsackie B virus infection according to laboratory tests.

Classification	Definition
Recent Infection	IgG and IgM negative previously with results showing seroconversion during pregnancy OR IgG and IgM positive, repeat test in 2 weeks with increasing IgG titers OR IgG negative with IgM positive, repeat test in 2 weeks and IgG becomes positive
Previous Infection	IgG positive with IgM negative OR IgG and IgM positive, repeat test in 2 weeks with plateau IgG titers
Non-immune (susceptive)	IgG and IgM negative
False Positive	IgG negative with IgM positive, test repeated in 2 weeks and IgG remains negative
Congenital Infection	PCR positive for parvovirus B19 in fetal blood collected by cordocentesis

## Data Availability

The data presented in this study are available on request from the corresponding author.
